# Antisense oligonucleotides targeting angiotensinogen: insights from animal studies

**DOI:** 10.1042/BSR20180201

**Published:** 2019-01-11

**Authors:** Chia-Hua Wu, Ya Wang, Murong Ma, Adam E. Mullick, Rosanne M. Crooke, Mark J. Graham, Alan Daugherty, Hong S. Lu

**Affiliations:** 1 Saha Cardiovascular Research Center, University of Kentucky, Lexington, KY, U.S.A.; 2 Department of Pharmacology and Nutritional Sciences, University of Kentucky, Lexington, KY, U.S.A.; 3 The Second Affiliated Hospital, Cardiovascular Key Laboratory of Zhejiang Province, Department of Cardiology, College of Medicine, Zhejiang University, Hangzhou, China; 4 Ionis Pharmaceuticals, Inc., Carlsbad, CA, U.S.A.; 5 Department of Physiology, University of Kentucky, Lexington, KY, U.S.A.

**Keywords:** antisense oligonucleotides, angiotensinogen, cardiovascular disease, hypertension

## Abstract

Angiotensinogen (AGT) is the unique substrate of all angiotensin peptides. We review the recent preclinical research of AGT antisense oligonucleotides (ASOs), a rapidly evolving therapeutic approach. The scope of the research findings not only opens doors for potentially new therapeutics of hypertension and many other diseases, but also provides insights into understanding critical physiological and pathophysiological roles mediated by AGT.

## Introduction

**Figure 1:**
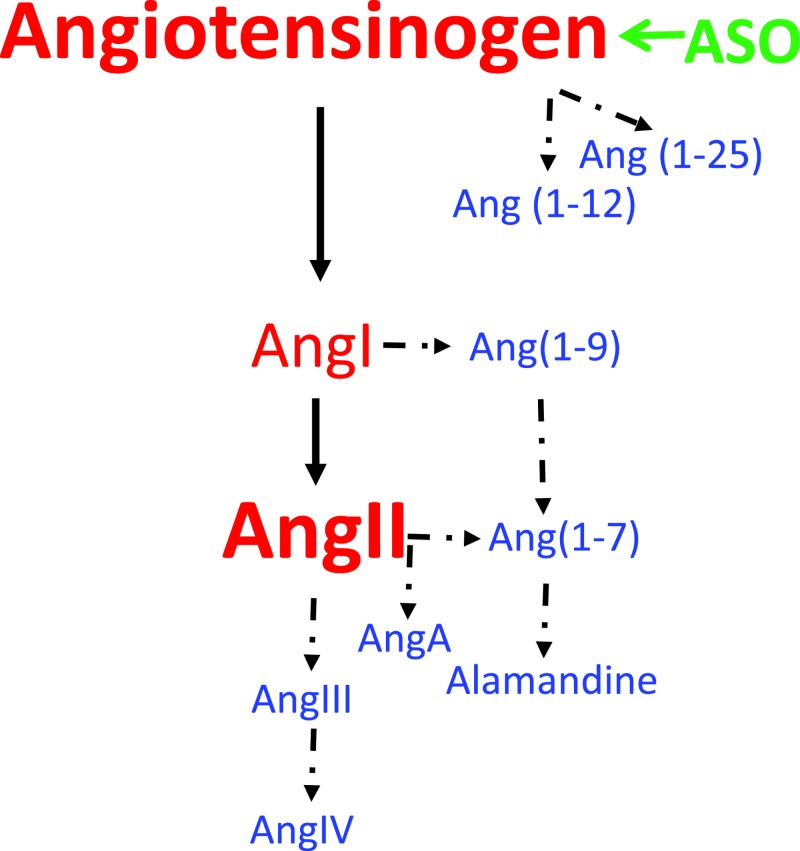
Angiotensinogen is the substrate of all angiotensin peptides ASOs targeting AGT inhibit AGT synthesis, thereby its downstream peptides. Solid arrows: classic components of the renin–angiotensin system. Dashed arrows: newly identified angiotensin peptides after 2000. Abbreviations: AGT, angiotensinogen; ASO, antisense oligonucleotide.

The renin–angiotensin system is an important regulator of many physiological and pathophysiological functions. Drugs inhibiting angiotensin-converting enzyme (ACE) to decrease angiotensin II (AngII) production or antagonizing AT1 receptor lower blood pressure and improve outcomes in patients with cardiovascular and renal diseases [1–4]. In addition to AngII, a spectrum of new bioactive angiotensin peptides (Figure 1) have been identified in the past two decades [5]. The biology of these angiotensin peptides has an evolving complexity with some of the peptides exerting opposing effects to AngII.

Angiotensinogen (AGT) is the only known substrate (Figure 1) of all angiotensin peptides [6,7]. Its pivotal roles have been demonstrated in mice with genetic whole-body deletion of AGT that have low neonatal survival rate, severe developmental problems, and low blood pressure [8,9]. The low viability and severe developmental issues of global AGT-deficient mice have led to a limited number of studies using this mouse model to explore its therapeutic potentials. Creation of AGT floxed mice has afforded the opportunity to understand the importance of tissue loci of AGT synthesis to its biological effects [10–12]. While these genetic approaches have the potential to provide insights into AGT biology, the interpretation of data derived from these mice needs to be tempered by their potential influences on embryonic development. Therapeutic inhibition of AGT mRNA expression using antisense oligonucleotides (ASOs) obviates developmental issues caused by genetic manipulations of AGT, thereby providing opportunities to more precisely determine roles of AGT in hypertension and many other diseases.

ASOs are single stranded, DNA-like synthetic nucleic acids designed to hybridize to specific RNAs or pre-mRNAs through Watson–Crick base pairing, ultimately modulating the production of a specific protein. Once this binding event occurs, there are multiple potential pathways through which ASOs mediate their pharmacological effects. A well-characterized mechanism results in the degradation of the RNA strand of a DNA–RNA duplex via the ubiquitously expressed cellular enzyme, RNase H1. ASOs can also reduce or increase gene expression through other mechanisms as well [13–15].

**Figure 2:**
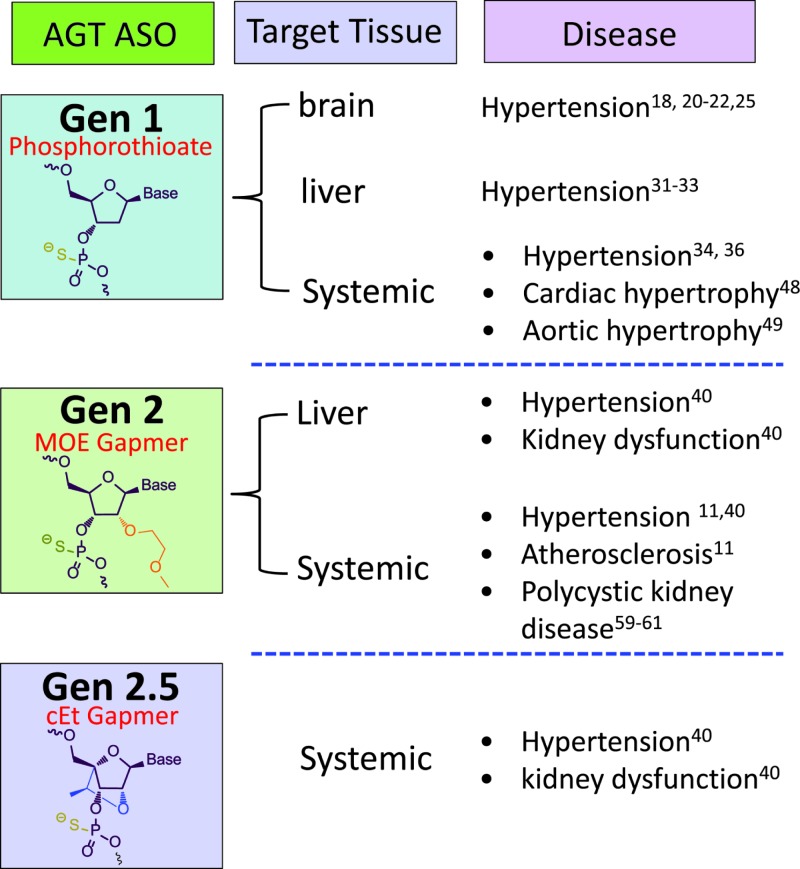
Summary of AGT ASO development and research in animal models from 1993 to 2017 ‘MOE Gapmer’ represents that ASO is flanked by MOE residues, which increases ASO binding affinity and RNase H cleavage at the center region of ASO. ‘cEt Gapmer’ represents that the ASO has 10–14 nucleotides central DNA region flanked by two to five of cEt residues, which improves hybridization with complementary sequences. Related representative references for each disease are denoted.

As natural DNA and RNA molecules are extremely labile due to susceptibility to endogenous nucleases, ASOs have been extensively altered to enhance their utility as therapeutic agents. ASOs modified by uniform deoxyphosphorothioates throughout the backbone are defined as generation 1 (Gen 1) [14]. The most widely used ASOs are chimeric molecules ranging from 13 to 20 nts in length that contain DNA-like 2′ deoxyphosphorothioates in the center region, with the termini modified in the 2′ position of the ribose moieties with methoxyethyls (MOE). This MOE ‘gapmer’-modified ASOs are defined as generation 2 (Gen 2). Generation 2.5 (Gen 2.5) ASOs have a similar ‘gapmer’ motif; however, the ribose is altered with a 2′,4′-constrained ethyl (cEt) design. These modified ASOs are more potent and stable than the first-generation drugs, and have increased duration of action allowing for more infrequent dosing and fewer unwanted side effects. The most advanced ASOs are conjugated to the hepatocyte targeting ligand (Triantennary N-acetylgalactosamine, ‘GalNAc’) and have been shown to improve ASO potency in liver by 10–30-fold and are currently under evaluation as a means to improve ASO safety, tolerability, and efficacy in several clinical programs [13–15]. The development of different generations of AGT ASOs and their applications to diseases in animal models are summarized in Figure 2.

This brief review will summarize animal studies using AGT ASOs, with the major focus on their antihypertensive effects.

## AGT ASO in animal models with high blood pressure

Effects of AGT ASOs on reducing hypertension have been studied in multiple animal models. Table 1 summarizes rat models that have been administered AGT ASOs. Collectively, these studies provide compelling evidence that inhibition of AGT synthesis by ASOs leads to profound reductions in blood pressure in hypertensive animal models.

**Table 1:** Effects of AGT ASO on blood pressure in hypertensive rat models

Animal Model	Sex	AGT ASO	Dose and duration	Route of injections	BP	References
SHR	Male	phosphorothioated 18-mers targeting bases −5 to +13 of *AGT* mRNA	50 μg/dose × 3 times at 12-h interval	i.c.v.	↓	[18,66]
			50 μg	i.c.v.	↓	[20]
				Carotid artery	↔	
			50 μg	Carotid artery	↓	[34]
		HVJ-liposome delivery (AGT ASO sequence: 5′ CTG CTT ACC TTT AGC T 3′)	N/A	Liver or portal vein	↓	[32]
		HVJ-liposome delivery (ASO targeting AGE2 in the promoter region)	7.5 nmol	Portal vein	↓	[33]
		HVJ-liposome delivery (ASO targeting AGE3 in the promoter region)	7.5 nmol	Portal vein	↔	
	N/A	ASO coupled to asialoglycoprotein carrier	10, 20, or 50 μg	tail vein	↓	[36]
	Male	Phosphorothioated 18-mers targeting bases −5 to +13 of *AGT* mRNA	0.2 nmol	p.v.n.	↔	[25]
			10 nmol	i.c.v.	↓	
SD rats 2K1C	Male	Phosphorothioated 18-mers targeting to bases -5 to +13 of *AGT* mRNA	50 μg	i.c.v.	↓	[22]
SD rats cold exposure	Male	Phosphorothioated 18-mers targeting to bases -5 to +13 of *AGT* mRNA	50 μg	i.c.v.	↓	[21]
			100 μg	Intracardiac	↓	
SHR high salt	Male	AGT ASO with 5-methyl modified cytosine and a phosphorothioate backbone containing either MOE or cEt modified sugars	5, 10, or 20 mg/kg	s.c.	↓	[40]

This table summarizes the reported studies: Administration of AGT ASO reduces blood pressure in hypertensive rat models. Hypertensive rat models included: (i) spontaneously hypertensive rats (SHR), (ii) 2-kidney, 1-clip (2K1C) surgery, (iii) cold exposure, and (iv) high salt diet. AGT ASO was administered through intracerebroventricular (i.c.v.), carotid arterial, liver, portal vein, tail vein, intracardiac, paraventricular hypothalamic nucleus (p.v.n.), or subcutaneous (s.c.) injection.

Abbreviation: **BP (blood pressure):** (1) ↓: decrease; (2) ↔: no change

**Injection route:** (1) i.c.v., intracerebroventricular; (2) i.p., intraperitoneal; (3) p.v.n., paraventricular hypothalamic nucleus; (4) s.c., subcutaneous.

**Rat models:** (1) SD: Sprague–Dawley rat; (2) SHR, spontaneously hypertensive rat; (3) 2K1C, 2K1C: 2-kidney, 1-clip hypertension.

**Others:** (1) AGE: AGT gene-activating elements; (2) HVJ: Hemagglutinating virus of Japan; (3) MOE (4) cEt.

### Targeting brain AGT

All of the components needed to produce AngII are present in the brain [16,17], which is recognized as an important tissue contributing to hypertension. The first study evaluating an AGT ASO for hypertension was reported by Gyurko and colleagues [18] using a well-established spontaneously hypertensive rat (SHR) model [19]. In this study, adult male SHR or normotensive control Wistar–Kyoto rats were administered an AGT ASO intracerebroventricularly (i.c.v.) via a cannula into the lateral ventricles. This led to reductions in systolic blood pressure for approximately 30–40 mmHg in SHR, but not in normotensive rats. Reductions in blood pressure in SHR were accompanied by decreased AngII concentrations in the brainstem, with no change in AngII concentrations in kidney or plasma [18]. In a subsequent study, a single i.c.v. injection of this AGT ASO resulted in a profound decrease in mean arterial pressure at 8 and 24 h post injection in SHR. In contrast, intra-arterial injection of this AGT ASO via a carotid artery catheter did not alter mean arterial pressure in SHR [20]. Further study found that i.c.v. injection of AGT ASO only reduced AGT concentrations in hypothalamus, but not in cortex, midbrain, and cerebellum. Blood pressure reductions produced by i.c.v. injection of AGT ASO was also supported by studies in male Sprague–Dawley rats in which hypertension was induced by either chronic exposure to a cold (5°C) environment [21] or a 2-kidney, 1-clip surgical procedure [22]. In agreement with these initial studies, administration of AGT ASO directly into the brain diminished hypothalamus AngII concentrations without changing plasma AngII [22]. These findings were consistent with AGT ASO reducing blood pressure by inhibiting AngII production locally within the brain.

Vasopressin is a hormone synthesized in the paraventricular hypothalamic nucleus, which is associated with AngII-mediated functions [23,24]. To explore whether inhibition of AGT synthesis via antisense technology reduced blood pressure through vasopressin-mediated mechanisms, AGT ASO was injected into the paraventricular hypothalamic nucleus of male SHR. This led to rapid reductions in plasma vasopressin and catecholamine (epinephrine and norepinephrine) concentrations, but did not change renin activity and mean arterial pressure [25]. However, this same dose of AGT ASO administered through i.c.v. injection reduced mean arterial pressure.

During the early development of the antisense technology platform, ASOs had low efficiency of uptake and short half-lives due to extensive, rapid endonuclease, and exonuclease degradation as we discussed earlier [13,26–28]. Although there are multiple shortcomings of the early AGT ASO drugs, there is accumulating evidence that (i) inhibition of AGT synthesis in brain by ASOs effectively reduces blood pressure in multiple hypertensive rat models, and (ii) this reduction in blood pressure is associated with changes in brain, but not plasma, AngII production, implicating local-specific effects of AngII in hypertension. Although i.c.v. injection is not readily adaptable to administer AGT ASOs in humans, these initial findings prompted efforts for the development of new generations of AGT ASOs with longer half-lives, improved potency and multiple routes of administration that would enable understanding the role of systemic AGT in hypertension.

### Targeting liver AGT

Liver is the major source of AGT in plasma [10,11]. Liver is also the major source of renal AGT protein under certain conditions such as in C57BL/6 mice fed a normal diet [12] and low-density lipoprotein (LDL) receptor^−/−^ mice fed a normal or Western diet [29]. Kidney, in addition to the brain, contributes to blood pressure regulation [30]. To determine effects of AGT in liver, Tomita and colleagues [31] used hemagglutinating virus of Japan (HVJ) containing liposome-encapsulated AGT ASO, and injected either directly into the liver or via the portal vein of male SHR. A single injection of AGT ASO led to pronounced decreases in hepatic and plasma AGT concentrations, plasma AngII concentrations, and blood pressure [31]. Blood pressure reductions were noted from days 1 to 4 with peak reduction at days 2 and 3, coincident with the most profound reductions in plasma AGT concentrations [32]. This effective inhibition of AGT synthesis leading to reductions in both plasma AGT concentrations and blood pressure only occurred when the AGT ASO targetted AGT gene-activating element 2 [33].

In addition to direct injections into the liver, administration of liposome-encapsulated AGT ASO synthesized as phosphorothioate modified 18-mers via a carotid artery catheter also led to rapid distribution of this ASO to liver [34]. This single injection significantly decreased mean arterial pressure, accompanied by reductions in plasma AGT and AngII concentrations. Inhibition of liver-derived AGT synthesis leading to reduced blood pressure was also demonstrated by a study with a single intracardiac injection of adeno-associated virus encoding full-length rat AGT to 5-day-old SHR, which reduced AGT expression in liver, delayed the onset of hypertension and decreased blood pressure for up to 6 months in adult SHR [35].

Makino and colleagues [36] administered asialoglycoprotein (ASGP)-poly(l)lysine-ASO complexes through tail vein injection in SHR, which decreased hepatic *AGT* mRNA abundance, plasma AGT and AngII concentrations, and blood pressure. However, ASGP-ASO was likely not targeting a specific hepatocyte receptor uptake pathway. Indeed as demonstrated recently with conjugation strategies targeting the ASGP receptor, ASGP-coupled AGT ASO was not directly targeting hepatocytes [37]. These data provide a proof-of-concept that systemic inhibition of AGT reduces blood pressure in hypertensive models, as would be expected given the significant role of AngII contributing to vascular and renal hypertensive mechanisms [38]. However, these studies used approaches that were suboptimal by today’s standards in regard to ASO potency and delivery [5].

With the rapid progress on antisense drug discovery technology [14], Gen 2.0 and 2.5 AGT ASOs were developed. One study used a hypercholesterolemic mouse model, LDL receptor^−/−^ mice, which have higher blood pressure than normolipidemic C57BL/6 mice [39]. Administration of Gen 2.0 AGT ASO intraperitoneally to LDL receptor^−/−^ mice profoundly reduced systolic blood pressure [11]. GalNAc–conjugated Gen 2.0 AGT ASO and unconjugated Gen 2.5 AGT ASO were evaluated in Wistar–Kyoto rats through subcutaneous injection [40]. GalNAc–conjugated AGT ASO did not reduce kidney AGT expression but led to profound reductions in liver-derived AGT and plasma AGT concentrations. Most importantly, GalNAc–conjugated AGT ASO significantly decreased blood pressure in SHR fed a diet containing 8% weight/weight salt. Although the mechanism has not been defined, there is consistent evidence that high salt diet suppresses the circulating renin–angiotensin system [41], which may contribute to diminished effects on blood pressure reduction by the renin–angiotensin inhibitors [42,43]. Therefore, SHR rats fed 8% salt diet is a hypertension model that is resistant to ACE inhibitors and AT1 receptor blockers, as also demonstrated in this study [40] using captopril and losartan, respectively. To evaluate the therapeutic index of avoiding the kidney with GalNAc, a renin–angiotensin–aldosterone challenge model of acute kidney injury generated via severe salt deprivation was evaluated. Kidney dysfunction was observed in salt-deprived rats administered captopril, losartan, or unconjugated Gen 2.5 AGT ASO. However, there was no evidence of renal dysfunction in animals administered the GalNAc–conjugated AGT ASO. Comparable results were observed in Sprague–Dawley rats with chronic kidney disease generated by 5/6 nephrectomy and fed the salt-depleted diet. Renal dysfunction or histopathology was not noted in animals injected with GalNAc–onjugated AGT ASO, whereas pathological effects were apparent in rats administered captopril or unconjugated Gen 2.5 AGT ASO [40]. These data provide evidence that AGT ASO targeting the liver might be superior to the classic renin–angiotensin inhibitors by maintaining antihypertensive efficacy but avoid impairing renal homeostatic functions dependent on AngII derived in part from kidney synthesized AGT. The renin–angiotensin system in circulation is suppressed by high salt diet or kidney injury, but its presence in kidney is activated [41]. Inhibition of AGT in liver blocks its supply to kidney, thereby diminishing renal AngII production [12]. The mechanisms whereby reductions in renal AngII by AGT ASO, but not by AT1a receptor blockade or ACE inhibition, lead to improved antihypertensive efficacy are unknown. Noncanonical renin–angiotensin enzymes and effectors, such as chymase and intracellular Ang II receptor signaling, may also contribute to the limited effectiveness of currently used renin–angiotensin inhibitors [5].

Overall, the published studies provide evidence that targeting AGT, as demonstrated using either earlier products of AGT ASO or the advanced Gen 2.0 and Gen 2.5 ASOs, has profound effects in reducing blood pressure. It is particularly interesting that targeting AGT in liver, but not systemic inhibition of AGT, ACE, or AT1 receptors, reduces blood pressure in a low renin–angiotensin rat model. It is also of potential therapeutic interest that inhibition of AGT synthesis in liver, but not globally, may be considered in patients who have not achieved sufficient blood pressure lowering on the standard of care or patients to whom renin–angiotensin blockade is contraindicated due to their poor kidney function. The extended duration of action of ASO therapy, typically requiring weekly or monthly dosing, will also be helpful to produce more constant renin–angiotensin blockade, especially beneficial in patients with poor compliance on the standard of care. Such a highly promising therapeutic profile will hopefully be realized by its ultimate application in patients.

## AGT ASO in animal models with other diseases

Although AGT ASOs have been studied extensively in hypertensive animal models, there are limited studies that determined their effects on other diseases. We review the few studies that report AGT ASOs on pulmonary fibrosis, cardiac or aortic hypertrophy, atherosclerosis, and polycystic kidney disease (PKD).

### Pulmonary fibrosis

Pulmonary fibrosis induced by bleomycin is associated with increased AGT and AngII concentrations in lungs of a rat model. In male Wistar rats, intratracheal injection of AGT ASO inhibited AGT synthesis and collagen accumulation in lungs, whereas serum AngII concentrations were not changed [44].

### Cardiovascular hypertrophy

AngII contributes to cardiac and aortic hypertrophy [45–47]. Tail vein injections of ASGP-coupled AGT ASO in male SHR from 10 to 20 weeks of age significantly inhibited cardiac hypertrophy [48] and aortic medial hypertrophy [49], accompanied by reductions in cardiac AT1 receptor mRNA, hepatic AGT mRNA as well as plasma AGT and AngII concentrations. However, intravenous administration of this AGT ASO did not change mRNA abundance of AGT, ACE, AT1, and AT2 receptors in the aorta [49].

### Atherosclerosis

Administration of Gen 2.0 AGT ASO intraperitoneally once a week prevented the development of atherosclerosis and slowed down its progression, but did not regress already established atherosclerotic lesions in LDL receptor^−/−^ mice [11]. AGT derived from liver is not only a target for hypertension, but also for many other cardiovascular diseases. In addition to administration of AGT ASO in LDL receptor^−/−^ mice, hepatocyte-specific AGT deficient mice were developed by breeding AGT floxed mice to transgenic mice having Cre recombinase under the control of the albumin promoter. Genetic deletion of AGT in hepatocytes ablated the development of atherosclerosis in LDL receptor-deficient mice fed a Western diet [10,11], demonstrating that hepatocyte-derived AGT contributes to atherosclerosis. AGT is the substrate to produce AngII, which interacts with its AT1a receptors to promoter atherosclerosis [7]. Whole body deficiency of AT1a receptor diminishes atherosclerosis [50–53], whereas depletion of AT1a receptor in endothelial cells or smooth muscle cells has no effects on atherosclerosis [54]. Therefore, AT1a receptor in vasculature is not the direct contributor to atherosclerosis. It also remains to be clarified where hepatocyte-derived AGT contributes to AngII production, activation of AT1a receptor, and thereby atherosclerosis.

### PKD

Mutations of PKD (PKD1 or PKD2) gene cause autosomal dominant PKD in humans [55]. AGT, renin, ACE, AngII, and AT1 receptors are present in cysts and dilated tubules of patients with PKD [56–58]. Three recent studies determined effects of AGT ASOs in PKD animal models [59–61]. Pkd2WS25/− or Pkd1^−/−^ mice were administered Gen 2.0 AGT ASO. Inhibition of AGT synthesis by this ASO was demonstrated by reduced *AGT* mRNA abundance in both liver and kidney as well as AGT protein in serum and kidney. In Pkd2WS25/− mice, administration of AGT ASO decreased kidney size, cyst volume density, and blood urea nitrogen, which were accompanied by reduced interstitial fibrosis in kidney, transforming growth factor-β, and proinflammatory cytokines CXCL1 and IL-12 [59]. In mice with global PKD1 deficiency, although lisinopril, an ACE inhibitor, led to more profound reductions in blood pressure, compared with AGT ASO, only AGT ASO attenuated kidney cyst formation [60]. Since blood pressure is considered as a sensitive marker for changes in AngII, we do not expect that AGT ASO inhibition be more effective in inhibiting AngII production than ACE inhibition. However, plasma and intrarenal AngII concentrations were not measured [60]. It is also unclear whether AGT ASO has more beneficial effects to PKD than other ACE inhibitors besides lisinopril and AT1 receptor blockers. In a subsequent study, accelerated renal pathology was induced by unilateral nephrectomy in mice with global PKD1 deficiency [61]. AGT ASO, with or without co-administration of aliskiren (a renin inhibitor), slowed progression of the cystic kidney pathology [61]. Unfortunately, this study did not report whether aliskiren alone would attenuate the progression of PKD.

The above studies provide evidence that AGT ASOs have profound beneficial effects on lung, heart, aorta, and kidney, although there are only one or a few studies reported for each organ or tissue in these animal models. However, to apply AGT ASOs to these diseases in human, consistent findings from different animal models will be needed. In these studies, only three articles reported both male and female animals, whereas the other studies only reported findings in male animals. Since sex differences have been reported in many of these diseases [62,63] and AGT is regulated by estrogen [64], it would be important to study both male and female animals before its application to human diseases.

## Clinical perspectives

Development of antisense therapy started 40 years ago [65] with the first ASO fomivirsen (Vitravene) approved by FDA in 1998 to treat patients with cytomegalovirus retinitis. Since then, another three ASOs have been approved by FDA and many more are in late stage development [13]. Although ACE inhibitors and AT1 receptor blockers provide extensive beneficial effects on hypertension and other cardiovascular diseases, there is still considerable interest in developing drugs that target the unique substrate of the renin–angiotensin system, with particular interest in developing ASOs since this therapeutic mode can be applied less frequently, while providing long lasting and highly specific control of blood pressure. Profound reductions in blood pressure and improvement of kidney pathologies by AGT ASO in hypertensive rat models with kidney dysfunctions [40] engenders confidence that AGT ASOs may have beneficial effects in addition to controlling blood pressure. It would also be expected that AGT ASOs targeting liver-specific AGT synthesis may have improved efficacy and safety on other AngII-mediated diseases such as heart failure, an area ripe for future discovery. A human AGT ASO developed by Ionis Pharmaceuticals, Inc. is under clinical evaluation in healthy volunteers (Clinical Trial NCT03101878). We look forward to clinical reports of this drug that has provided consistent and profound beneficial effects in multiple animal models.

## Abbreviations

ACE: angiotensin-converting enzyme
AGT: angiotensinogen
AngII: angiotensin II
ASGP: asialoglycoprotein
ASO: antisense oligonucleotide
cEt: 2′,4′-constrained ethyl
i.c.v.: intracerebroventricular
LDL: low-density lipoprotein
MOE: methoxyethyl
PKD: polycystic kidney disease
SHR: spontaneously hypertensive rat
